# Standard vs. Nutrient-Enriched Cow’s Milk and Its Impacts on Child Growth: A Systematic Review and Meta-Analysis

**DOI:** 10.3390/nu15051124

**Published:** 2023-02-23

**Authors:** Darwish Mohd Isa, Raanita Krishnamoorthy, Hazreen Abdul Majid

**Affiliations:** 1 Centre for Population Health, Department of Social and Preventive Medicine, Faculty of Medicine, University of Malaya, Kuala Lumpur 50603, Malaysia; 2 School of Chiropractor, AECC University College, Parkwood Campus, Parkwood Road, Bournemouth, Dorset BH5 2DF, UK

**Keywords:** cow’s milk, children, growth, systematic review, meta-analysis

## Abstract

Stunting among children indicates malnutrition or undernutrition, hindering their growth and development. This will have negative effects on the overall health of children. This review investigates the effects of different types of cow’s milk and their impacts on children’s growth. A web-based search of Cochrane, Web of Science, SAGE, and Prospero was carried out using predetermined search/MESH phrases and keywords. The data extraction and analysis were carried out independently by two reviewers, who then double-checked, revised, and discussed any disagreements with a third reviewer. Eight studies met the inclusion criteria and were rated as good (N = 5) and fair quality (N = 3), which were included in the final analysis. The results illustrated that standard cow’s milk has more consistent findings than nutrient-enriched cow’s milk potentially in assisting children’s growth. However, studies on standard cow’s milk and child’s growth are still lacking for this age group. In addition, there are inconsistent findings between nutrient-enriched cow’s milk and children’s growth. It is crucial to ensure milk is included in children’s diets as per recommended nutrient intake.

## 1. Introduction

Stunting is one of the main indicators of childhood malnutrition. It signifies that a child has failed to reach their growth potential due to illness, poor health, and malnutrition [1]. A child is considered ‘stunted’ if they are too short for their age or height-for-age is more than two standard deviations below the WHO Child Growth Standards median [2], implying that their growth and development are hindered. 

Globally, more than one in five children is stunted. Stunting has steadily declined since 2000; it was recorded at over 30% and declined to around 20% in the year 2020, affecting 200 million and 140 million children, respectively [3]. Nevertheless, faster progress is needed to reach the global target by 2030. Undernutrition causes about half of all fatalities in children under the age of five, making them more regular and severe and slowing their ability to recover [2].

Based on the current literature across 33 countries, at least 30% of children are still affected by stunted growth [3]. Based on published research among children six years and above, stunting prevalence is 57% among primary school children in Ethiopia and 50.3% in Ghana, which is alarming [4,5]. In the Middle East and North Africa region, the stunting prevalence is 16.5%, followed by Nigeria, 17.4% [6,7]. Children in the rural parts of North India and North Sri Lanka have a stunting prevalence of 9.25% and 11.3%, respectively. Dairy products and cow’s milk promote linear growth in children, resulting in taller adults [8]. High-quality proteins, bioavailable growth-promoting minerals, and possibly lactose are all components of cow’s milk that are considered to aid growth. In many countries, milk is recommended in daily diet, ranging from 2–3 cups every day [9,10,11]. Numerous meta-analyses have found neutral or positive relationships between total dairy and milk consumption and body weight and fat mass levels [12,13], glycaemic and lipidemic profiles [14,15], blood pressure, and cardiorespiratory fitness indices across all age groups [16], as well as their optimal skeletal growth and development [10].

There are many beneficial effects of consuming cow’s milk or dairy products. However, there is still a lack of studies confirming the effects of different types of cow’s milk on children’s growth and development. This systematic review aims to determine the relationship between different types of cow’s milk (standard vs. nutrient-enriched) and how it may impact children’s growth. 

Standard milk, also known as adjusted milk, is milk that has had the original fat content and the ratio of fat to other milk solids modified. This can be done by removing milk fat, adding skim milk, or adding cream [17]. This includes primary available milk types such as whole or plain milk, reduced-fat milk, low-fat milk, and fat-free milk. Nutrient-enriched or fortified milk is milk that contains extra vitamins, typically vitamins A and D [18], or other various nutrients, such as zinc, iron, and folic acid [19], which can usually be found in formula milk. This systematic review will identify the impacts of both types of cow’s milk on a child’s growth.

## 2. Materials and Methods

### 2.1. Eligibility Criteria

Preferred Reporting Items for Systematic Reviews and Meta-Analyses (PRISMA 2020) reporting guidelines and PICOS (Participant, Intervention, Comparison, Outcomes, and Study Design) criteria were used to conduct the systematic review (Table 1). The International Prospective Register of Systematic Reviews has documented the review procedure (PROSPERO Registration number. CRD42022355870).

Research that discussed the links between milk type and its effects on children ages 7 to 12 years old was identified through a thorough literature search. It centred on the link between milk type and child growth, measuring growth with quantitatively validated tools. Only direct interventions intended to enhance child growth were considered for intervention trials. Studies that did not employ the proper tools, provided no related results, were of poor quality, featured patients with certain conditions, or were not published in English were all excluded.

### 2.2. Information Sources

The following databases were searched on the internet: Cochrane, Web of Science, SAGE, and Prospero. In addition to manual searches, database searches were conducted on grey literature, the internet (such as Google Scholar), reference lists of works cited in systematic reviews, and other sources. On 1 December 2022, the last search was conducted.

### 2.3. Search Strategy

The search was restricted to English-language publications published between December 2000 and December 2022. Another search was conducted again before the final analysis. A combination of keywords and MESH terms, including “milk” OR “fresh milk” OR “milk powder” OR “liquid milk” AND “stunting” OR “growth” OR “stunted” AND “child” OR “children” were used in the search (Table 2).

### 2.4. Selection Process

Three stages were followed in the selection process for the articles: title selection, review of the abstract, and full-text evaluation. Author, publication year, title, journal, inclusion and exclusion criteria, subject recruitment, age, gender, research duration, and dates were all retrieved along with study design, setting, and country information.

### 2.5. Data Collection Process

The collected information was then exported to Mendeley, a reference manager software, and Microsoft Excel for data extraction. The review process was conducted on Rayyan, a website and a review tool where both authors can independently perform the data extraction and analysis, cross-check, and have a third reviewer examine and resolve any differences.

### 2.6. Data Items

The review included methods for assessing subjects’ stunting, the types of cow’s milk used, and the associations’ results to understand the links between milk type and child growth. Additional factors were also retrieved, including sociodemographic characteristics, bone mineral density, and nutritional status. The results were then documented, along with the conclusions and suggestions drawn from them using statistical methods. In every study, we looked for outcomes that fit the outcome domain. When further information regarding the study results or other specific data was required, the studies’ original authors were contacted.

### 2.7. Study Risk of Bias Assessment

Observational studies’ selection, comparability, and results were evaluated using the Newcastle-Ottawa quality assessment scale, which was modified for cross-sectional and cohort studies. The Cochrane Collaboration Tool was used to evaluate the random sequence generation, allocation concealment, selective reporting, other bias, blinding of participants and staff, blinding of outcome assessment, and missing outcome data for intervention studies. The quality ratings ranged from “Good”, “Fair”, to “Poor”. Two reviewers separately evaluated the potential for bias in each paper, and any discrepancies were discussed with the third author. The tabulation included information about the bias evaluation.

### 2.8. Outcome Measures

Effective measures, including mean, mean difference, and odds ratios of the outcomes, were utilised to synthesise and display results after gathering binary and continuous outcomes. Quartiles and other calculations and statistics were also included in the review.

### 2.9. Synthesis Methods

The results were tabulated and presented visually. The relationship between the type of cow’s milk and child growth was summarised narratively.

## 3. Results

### 3.1. Study Selection

**Figure 1:**
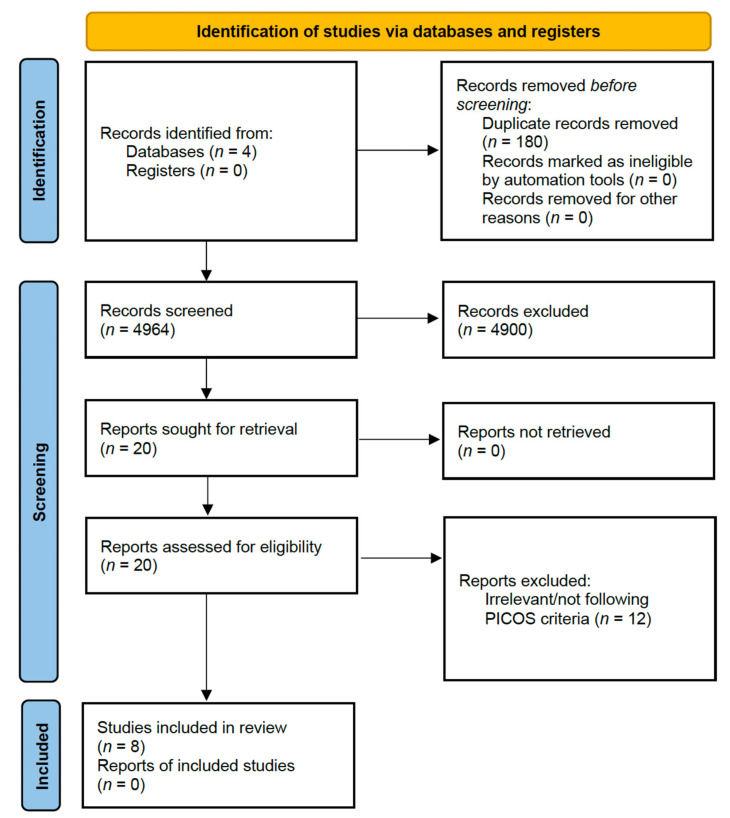
PRISMA flow diagram of the study.

Four thousand nine hundred eighty-four articles were found after duplicate databases entries were removed. Following the screening of the title and abstract, 20 full texts were evaluated. Eight studies out of 20 fulfilled the criteria for inclusion, while 12 were excluded as they involved infants and did not meet the criteria or were irrelevant to the objectives. Five papers had good quality, and three had fair quality after the quality assessment. All eight articles were included in the synthesis. Figure 1 displays the PRISMA flow diagram for the literature search.

### 3.2. Study Characteristics

Table 3 demonstrate the study characteristics of the sampled studies. Three cross-sectional studies, one cohort study, and four RCTs were included in the eight papers. These studies were conducted in the United States (N = 7), China (N = 2), the United Kingdom (N = 2), India (N = 1), and Greece (N = 1) [20,21,22,23,24]. Out of all the studies, five studies show a significant positive association between milk intake and growth indicators such as height and sitting height [20,21,22,25,26]. In comparison, two studies show a significant negative association [23,24]. 

All studies used validated instruments to assess the child’s growth for the outcome measurement. Nevertheless, the measurement parameters that were used to compare the growth results between the treatment and control groups varied. Sitting height and body mass index was measured across three studies in China [20,21,22]. Three studies used the validated Dual Energy X-ray Absorptiometry (DXA) technology to determine the Bone Mineral Density (BMD) at various skeletal sites [20,21,22]. It is interesting to note that the study by Zhu et al. (2005) showed a significant increase in the combined cortical thickness (CCT) in the intervention group after 24 months compared to the control group [20]. The measurement of CCT is the best method of diagnosing osteoporosis quantitatively. Similarly, Du et al. (2004) documented those subjects who received additional cholecalciferol than those who received milk without added cholecalciferol had significantly greater increases in the total-body bone mineral content (BMC) (2.4% vs 1.2%) and BMD (5.5% vs 3.2%) [21]. In contrast, Zhu et al. (2006) study demonstrated no significant differences between the groups’ total-body BMC and BMD changes. However, the intervention group receiving calcium-fortified milk experienced substantially higher increases in sitting height than the control group (0.9 ± 0.3%; *p* = 0.02, *p* < 0.05) [20].

### 3.3. Quality Assessment

Five of the eight papers included are rated as good, while the other three are of fair quality. Table 4, Table 5 and Table 6 for cross-sectional studies, cohort studies, and randomised trials, respectively, show a detailed summary of quality appraisal.

**Table 3:** (**a**) General characteristics of the studies included and their outcomes for observational studies. (**b**) General characteristics of the studies included and their outcomes for randomised controlled trials.

Author (Year)	Design	Outcome Instrument	Quality Score	Study Sample	Outcomes
(a)					
Moschonis et al. (2016) [24] Greece	Cross-sectional study	BMI, Height, Endurance run test (ERT)	Good	600	Milk consumption had a positive correlation (β = 0.10; *p* = 0.017) with the number of ERT stages completed and a negative correlation (β = −0.10; *p* = 0.014) with body mass index (BMI). Type of milk intake: Formula milk
Guo et al. (2020) [26] China	Cross-sectional study	Weight, Height, BMI	Good	40,607	After adjusting for confounding factors, the low- and high-intake groups for girls were 0.83 cm (95% confidence interval: 0.00, 1.68 cm) and 1.26 cm (0.34, 2.19 cm) taller than the no-intake group, respectively. Boys who consumed more milk than those who did not have lower BMIs (−0.56, 95% CI: −1.00, −0.12 kg/m^2^) and a decreased risk of obesity (OR = 0.67, 95% CI: 0.46, 0.97). Type of milk intake: Questionnaire which includes classification of milk intake as - Plain milk/yogurt - Plain whole, low-fat, skim cow’s milk
Wiley et al. (2005) [23] USA	Cross-sectional study	Height	Good	2592	After considering factors such as age, birthweight, energy intake, and ethnicity, milk consumption did not affect the height of children aged 5 to 11 years. Type of milk intake: Formula milk
Hopkins et al. (2015) [25] UK	Cohort	Weight, Height, and BMI	Good	1112	From 8 months to 10 years of age, children in the cow’s milk group were heavier than breastfed children, with weight differences of ≥0.27 SD scores (SDSs) and an average of 0.48 SDSs (after adjusting for maternal education, smoking, and parity). At 18 months of age, there was a maximum weight difference (0.70 SDS; 95% CI: 0.41, 1.00 SDS; *p* = < 0.0001). Children in the cow’s milk group had greater BMI SDSs starting at age 8 months (at age 9; *p* = 0.001) and were taller at some ages (25–43 mo; *p* = 0.01). From 8 to 37 months of age, children in the formula milk group were heavier and taller than breastfed children. At 8 months of age, there were obvious dietary differences between the milk groups, some of which persisted to 18 months. The differences in growth that were observed were not attenuated by adjusting for current protein and energy intakes. Type of milk intake: Cow’s or Formula milk
(b)					
Kuriyan et al. (2016) [27] India	Randomised control trial	Anthropometry, Height, Cognitive measures, Physical endurance and Agility, Blood biochemistry.	Good	224	Anthropometry: Children’s mean height changes in the control group (3.7 ± 0.8 cm) and the intervention group (4.0 ± 0.9 cm) both exhibited a trend towards significance, with the intervention group showing a stronger effect (*p* = 0.07). Cognitive and physical performance: Most of the cognitive measures in the paired analyses of morning and evening showed a significant improvement from baseline to end line (*p* < 0.01), except for Trial A, which had no correct answers for the evening assessment in the control group, and Trial B, which had no correct answers for the evening assessment in both groups. At the end of this study, both groups improved their physical endurance and agility, and there was no significant interaction between the study groups regarding time or status. Type of milk intake: Formula milk
Zhu et al. (2005) [20]	Randomised control trial	Metacarpal morphometry and bone age, biochemical analysis, Body weight, Height, Sitting height, BMC, Bone area, BMD of total body and forearm, dietary intakes, Breast, and pubic hair development, and Date of menarche.	Fair	606 girls with complete hand X-ray radiographs and 128 girls with complete biochemical analysis.	Subjects in all 3 groups were noticeably heavier and taller at 24-mo compared to baseline. After adjusting for pubertal status and school clustering, 24-mo supplementation resulted in larger gains in periosteal diameter (1.2%) and cortical thickness (5.7%) compared to the control, but smaller gains in medullary diameter (6.7%) (*p* < 0.05). In comparison to the control group, the calcium and vitamin D–fortified milk (CaD) milk group had lower serum bone alkaline phosphatase (BAP) at 12 mo (19.9%) and parathyroid hormone (PTH) at 12 mo (46.2%) and 24 mo (16.4%). (*p* < 0.05). After clustering by the school was considered, the effect of milk supplementation on increasing Insulin-like growth factor I (IGF-I) concentrations at 24 mo (16.7–23.3%) were no longer significant. Increases in periosteal diameter, CCT, and second metacarpal length were observed in all 3 groups of girls. Contrarily, over 24 months, medullary diameter increased in the control group, decreased in the Ca milk group, and remained unchanged in the CaD milk group. After adjusting for baseline value, bone age, Tanner breast, and pubic hair development stage, menarcheal status at 24 mo, and clustering by the school, supplementation had resulted in significantly greater increases in periosteal diameter, CCT, and length of the second metacarpal after 24 mo compared to the control intervention and significantly smaller gains in medullary diameter. Periosteal diameter changes were correlated positively with total-body BMC changes (r = 0.418, *p* < 0.001), bone area changes (r = 0.233, *p* < 0.001), and BMD changes (r = 0.139, *p* = 0.015). Percentage changes in medullary diameter were negatively correlated with those of total-body BMC (r = −0.163, *p* = 0.004) and BMD (r = 0.234, *p* < 0.001). Percentage changes in CCT were positively correlated with those of total-body BMC (r = 0.329, *p* < 0.001) and BMD (r = 0.251, *p* < 0.001). Type of milk intake: Formula milk
Zhu et al. (2006) [22] China	Randomised control trial	Bone area, body weight, and height	Fair	587	Between the groups, there were no significant differences in the total-body BMC and BMD changes since baseline. Sitting height increased significantly in the calcium-fortified milk group (0.9 ± 0.3%; *p* = 0.02) than in the control group. The serum 25-hydroxyvitamin D concentrations of the group receiving calcium- and vitamin D-fortified milk were 17.1 6.7% lower than those of the control group (*p* = 0.04), but this difference was reduced by adjusting for physical activity level (14.2 ± 6.7%; *p* = 0.08). Type of milk intake: Formula milk
Du et al. (2004) [21] China	Randomised control trial	The distal forearm of the non-dominant arm, Proximal forearm of the non-dominant arm, Total body, BMC, Bone area (BA), BMD, Height, Sitting height, and Weight.	Fair	698	With or without the addition of cholecalciferol, two years of milk consumption resulted in significantly bigger changes in height (≥0.6%), sitting height (≥0.8%), body weight (≥2.9%), total-body BMC (≥1.2%), and BMD (≥3.2%). The change in total-body BMC (2.4% vs. 1.2%) and BMD (5.5% vs. 3.2%) was significantly higher in subjects who received additional cholecalciferol than in subjects who received milk without added cholecalciferol. Type of milk intake: Formula milk

CCT: Combined cortical thickness; SDSs: Standard Deviation scores; RCF: Red cell folate; BMI: Body-mass-index.

### 3.4. Meta-Analysis

**Figure 2:**
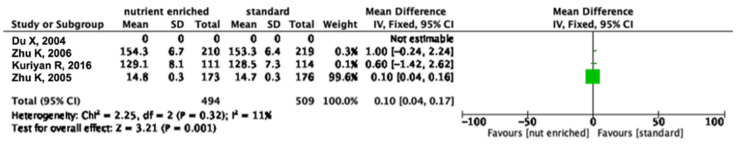
Meta-analysis of studies [20,21,22,27].

The I² test shows 11%, hence showing a low heterogeneity (Figure 2). The studies’ overall odds ratio (OR) is 0.10, which falls within the confidence interval range of 0.04 to 0.17, which is statistically significant, and the result is interpretable. The results of the OR are in favour of drinking standard cow’s milk rather than drinking nutrient-enriched cow’s milk towards growth. However, there is insufficient evidence to conclude the findings, and there were only three studies applicable for meta-analysis. More studies that include mean and standard deviation are required for more accurate results.

### 3.5. Different Types of Cow’s Milk and Height

The studies used two types of cow’s milk as the intervention/exposure, namely nutrient-enriched and standard cow’s milk (Table 7). There are inconsistent findings between nutrient-enriched cow’s milk and the outcomes. Overall, most of the studies included have shown a significant positive association between milk and height [20,21,22,25,26], two significant negative associations [23,24], and one insignificant association [27].

## 4. Discussion

Growth and development during childhood are crucial. The double burden of childhood malnutrition is a worldwide issue. There may be some noticeable differences in children’s height, weight, and build during the school-age child development phase [28]. Undernutrition severely impairs children’s physical, mental, and cognitive abilities and places a strain on families, communities, and nations [29]. In addition, childhood growth and weight increase are significant indicators of dietary intake, nutritional status, and physical development. Furthermore, a childhood BMI that was higher than normal has also been linked to a higher chance of developing coronary heart disease as an adult [30,31]. Out of eight papers that were included in the review, most of them (N = 5) found positive associations between cow’s milk and height. This review discusses the impact of different types of cow’s milk and how it impacts children’s growth and development.

Nutrient-enriched and standard cow’s milk are highly nutritious and rich in vitamin B12, calcium, and phosphorus. However, nutrient-enriched cow’s milk can be fortified with various nutrients, typically vitamins D and A [18], zinc, iron, and folic acid [19]. Depending on where we live and what nutrients might be lacking in our diet, milk may or may not be fortified. While other nations have laws requiring milk fortification, this is not the situation in the United States [32]. However, despite the need to supplement the children with milk, it is crucial to include other healthier choices of foods as a holistic approach to their overall dietary recommendations.

Most studies included in this review used nutrient-enriched cow’s milk or formula milk for their participants and found positive impacts on the children’s growth, such as height. A study conducted in China demonstrated that the low- and high-milk-intake groups were taller than the no-intake groups for girls, and boys with high milk intake had lower BMI and risk of obesity [26]. These findings were parallel with other studies [20,21,22,25]. Another study showed a positive association, but the result was non-significant despite the *p*-value being close to the significance level (*p* = 0.07, *p* > 0.05) [27].

A few conflicting findings should be highlighted. A cross-sectional study conducted in Greece reported that milk consumption was associated negatively with BMI (β = −0.10; *p* = 0.014) [24]. Another cross-sectional study conducted in the United States showed that there is no impact between milk consumption and height after controlling for age, birthweight, energy intake, and ethnicity. This may be due to the nature of cross-sectional studies, which makes it difficult to make a causal inference [33]. However, according to the “Children of 1997” birth cohort, which included many children, “milk intake and other dairy products were not linked with BMI z-score among children with milk intake at 1–3 times/day” (Adjusted = 0.01, 95% CI: 0.04–0.06). This study suggested that to determine whether observed correlations are physiologically mediated or socially confounded, evidence from non-Western developed cultures with diverse social patterns is important [34]. All studies using standard cow’s milk in this review found significant positive associations between the milk ingested and height. Nevertheless, more studies are required to further provide more accuracy in these findings.

There are several limitations to increasing milk consumption among children, for example, in certain conditions, such as milk allergy and lactose intolerance, which are often seen among children and young adults; in fact, 65% of the world’s population is lactose intolerant [35]. These are the conditions where their milk intake is usually compromised. Some people who are lactose intolerant can handle certain types of milk and milk-containing products, so they might not need to be avoided. It is common to misunderstand the difference between lactose intolerance and milk allergy. While lactose intolerance is a gastrointestinal disorder, milk allergy is an immunological reaction to milk proteins [36]. This suggests using other alternatives or sources to ensure children with such conditions achieve optimal growth.

Calcium is essential for the development of bones and the skeleton [37]. The recommended daily calcium intake for children aged 7 to 12 ranged from 1000 to 1300 mg/day [31]. To date, there are still inconsistent findings regarding the association between calcium and linear growth. A cohort study in China [38] revealed that for boys who consume plant-based diets, increasing calcium intake throughout adolescence is associated with faster height growth but not adult height; calcium intake below 300 mg/d may result in shorter adult stature. However, no significant associations were found in girls. Another study conducted among five- to fifteen-year-old girls reported that height Z-scores were significantly related to calcium absorption [39]. It is therefore important to highlight the credible effects of calcium on growth which can be consumed through dietary sources and calcium supplements through proper prescriptions. 

A study conducted in China to promote dairy milk and food intake found that preschool children who did not consume dairy were 1.03 times more likely to experience stunting than those who consumed dairy milk (AOR: 1.03, 95% CI: 0.74–1.42) [26]. When compared to children who develop normally, stunting might result in the loss of two to three years of schooling and a subsequent 23% income reduction in adulthood [40]. Additionally, it appears to be linked to a higher chance of developing chronic, non-communicable diseases like diabetes, hypertension, and cardiovascular disease [41]. Low dairy consumption is still common among low- and middle-income countries due to low affordability, accessibility, and availability [42]. According to a systematic review of controlled trials, physiology supports the consumption of milk and dairy products during the paediatric period because they have the essential nutrients for growth and development [43,44].

## 5. Conclusions

Stunting or undernutrition may have a greater impact on children’s growth. By comparing both types of milk, it appears that standard milk has shown more consistent findings potentially in assisting growth among children aged 7–12 years old ; further intervention research looking into this area is required. It is still important to include milk in children’s diet as per recommended nutrient intake to support their growth, regardless of the type.

## Figures and Tables

**Table 1:** PICOS Criteria.

Criteria	Description
Participants	Children aged between 7–12 years old
Intervention/Exposure	Different types of cow’s milk
Comparison	Standard vs Nutrient-enriched cow’s milk
Outcomes	Child’s growth using validated measurements and protocol
Study design	Randomised controlled trial (RCT), non-RCT, cohort, and cross-sectional

**Table 2:** Search Strategy.

Concept 1		Concept 2		Concept 3
(“Milk” [All fields] OR “Fresh Milk” [All fields] OR “Milk powder” [All fields] OR “Liquid milk”[All Fields])	AND	(“Stunting” [All fields] OR “Growth”[All fields] OR “Stunted” [All Fields])	AND	(“child”[MeSH Terms] OR “children” [MeSH Terms])

**Table 4:** Quality assessment of cross-sectional studies.

Studies/Domains	Selection				Comparability	Outcome			
	Representativeness of the Sample	Sample Size	Non-Respondents	Ascertainment of Risk Exposure	Comparability	Assessment of Outcome	Statistical Test	Selection—Score+Comparability Score+Outcome	Quality
Moschonis et al., 2018 [24]	*	*	*	**	**	*	*	5 + 2 + 2 = 9	Good
Guo et al., 2020 [26]	*	*	*	**	*	*	*	5 + 1 + 2 = 8	Good
Wiley, 2005 [23]	**	*	*	**	**	*	*	5 + 2 + 3	Good

Good quality: 3 or 4 stars in the selection domain, 1 or 2 two stars in the comparability domain, and 2 or 3 stars in the outcome/exposure domain. Fair quality: 2 stars in the selection domain, 1 or 2 stars in the comparability domain, and 2 or 3 stars in the outcome/exposure domain. Poor quality: 0 or 1 star in the selection domain, 0 stars in the comparability domain, and 0 or 1 star in the outcome/exposure domain.

**Table 5:** Quality assessment of cohort studies.

Studies/Domains	Selection				Comparability	Outcome				
	Representativeness of the Exposed Cohort	Selection of the Non-Exposed Cohort	Ascertainment of Exposure	Demonstration that Outcome of Interest was not Present at the Start of the Study	Comparability	Assessment of Outcome	Was Follow-Up Long Enough for Outcomes to Occur	Adequacy of Follow-Up of Cohorts	Selection Score+Comparability Score+Outcome	Quality
Hopkins et al., 2015 [25]	*	*	*	*	*	*	*	*	4 + 1 + 3 = 8	Good

Good quality: 3 or 4 stars in selection domain AND 1 or 2 stars in comparability domain AND 2 or 3 stars in outcome/exposure domain. Fair quality: 2 stars in selection domain AND 1 or 2 stars in comparability domain AND 2 or 3 stars in outcome/exposure domain. Poor quality: 0 or 1 star in selection domain OR 0 stars in comparability OR 0 or stars in outcome/exposure domain.

**Table 6:** Quality assessment of randomised controlled studies.

Studies	Random Sequence Generation	Allocation Concealment	Blinding Of Participants and Personnel	Blinding of Outcome Assessment	Incomplete Outcome Data	Selective Reporting	Other Bias Due to Problems Not Covered Elsewhere in the Table	Quality Score—Good Quality/Fair Quality/Poor Quality
Zhu et al., 2006 [22]	High Risk	Low Risk	Low Risk	Low Risk	Low Risk	Low Risk	Unclear	Fair
Du et al., 2004 [21]	High Risk	Low Risk	Low Risk	Low Risk	Low Risk	Low Risk	Unclear	Fair
Kuriyan et al., 2016 [27]	Low Risk	Low Risk	Low Risk	Low Risk	Low Risk	Low Risk	Low Risk	Good
Zhu et al., 2005 [20]	High Risk	Low Risk	Low Risk	Low Risk	Low Risk	Low Risk	Unclear	Fair

Good quality: All criteria met (i.e., low for each domain) Fair quality: One criterion not met (i.e., high risk of bias for one domain) or two criteria unclear, and the assessment that this was unlikely to have biased the outcome, and there is no known important limitation that could invalidate the results Poor quality: One criterion not met (i.e., high risk of bias for one domain) or two criteria unclear, and the assessment that this was likely to have biased the outcome, and there are important limitations that could invalidate the results Poor quality: Two or more criteria listed as high or unclear risk of bias.

**Table 7:** Different types of milk and the outcomes.

Type of Milk	Design (Total Number of Studies by Design)	Positive Results (*p* < 0.05)	Negative Results (*p* < 0.05)	Non-Significant Results (*p* > 0.05)
Nutrient-enriched cow’s milk/Formula milk	Cross-Sectional (N = 2) Cohort (N = 1) Randomised Controlled Trial (N = 4)	4	2	1
Standard cow’s milk/Plain milk, and other dairy products	Cross-Sectional (N = 1) Cohort (N = 1) Randomised Controlled Trial (N = 0)	2	0	0

## Data Availability

Data are contained within the article.
